# Dissection of a metastatic gene expression signature into distinct components

**DOI:** 10.1186/gb-2006-7-12-r117

**Published:** 2006-12-11

**Authors:** Paul Roepman, Erica de Koning, Dik van Leenen, Roel A de Weger, J Alain Kummer, Piet J Slootweg, Frank CP Holstege

**Affiliations:** 1 Department of Physiological Chemistry, University Medical Center Utrecht, Universiteitsweg, Utrecht, the Netherlands; 2 Department of Pathology, University Medical Center Utrecht, Heidelberglaan, Utrecht, the Netherlands

## Abstract

**Background:**

Metastasis, the process whereby cancer cells spread, is in part caused by an incompletely understood interplay between cancer cells and the surrounding stroma. Gene expression studies typically analyze samples containing tumor cells and stroma. Samples with less than 50% tumor cells are generally excluded, thereby reducing the number of patients that can benefit from clinically relevant signatures.

**Results:**

For a head-neck squamous cell carcinoma (HNSCC) primary tumor expression signature that predicts the presence of lymph node metastasis, we first show that reduced proportions of tumor cells results in decreased predictive accuracy. To determine the influence of stroma on the predictive signature and to investigate the interaction between tumor cells and the surrounding microenvironment, we used laser capture microdissection to divide the metastatic signature into six distinct components based on tumor versus stroma expression and on association with the metastatic phenotype. A strikingly skewed distribution of metastasis associated genes is revealed.

**Conclusion:**

Dissection of predictive signatures into different components has implications for design of expression signatures and for our understanding of the metastatic process. Compared to primary tumors that have not formed metastases, primary HNSCC tumors that have metastasized are characterized by predominant down-regulation of tumor cell specific genes and exclusive up-regulation of stromal cell specific genes. The skewed distribution agrees with poor signature performance on samples that contain less than 50% tumor cells. Methods for reducing tumor composition bias that lead to greater predictive accuracy and an increase in the types of samples that can be included are presented.

## Abstract 2

The dissection of predictive expression signatures into different components based on tumor versus stroma expression shows a strikingly skewed distribution of metastasis associated genes.

## Background

DNA microarray technology has advanced our understanding of cancer by providing genome-wide mRNA expression measurements of different tumor types [1-3]. Such studies have been used to identify new subtypes of cancer [4-7]. Specific gene expression signatures have been found that can predict treatment response [8], metastatic disease [9,10], and recurrence rate [11] and that are associated with poor outcome in cancer patients [12,13]. Despite the fact that some aspects of signature discovery studies still need optimization [14-16], the potential of cancer genomics is already starting to be realized, with the first signatures becoming available for use in the clinic or in their final prospective validation phase [17].

Although in a few cases laser capture microdissection (LCM) has been applied [18,19], expression profiling studies of solid tumors generally employ whole tumor sections consisting of tumor cells and the surrounding tissue microenvironment. This includes extracellular matrix components and stromal cells, such as fibroblasts and immune response cells [20]. Because gene expression patterns are thus derived from both tumor cells and tumor stroma, it is important to consider the degree to which inclusion of stromal cells influences the outcome of tumor profiling studies.

This general question is particularly interesting when considering signatures for prediction of metastasis. Metastasis is the process whereby cancer cells spread to other sites in the body and is the principal cause of cancer-related deaths. To choose appropriate treatment strategies, it is of great importance to assess the presence of metastasis in cancer patients [21]. It has recently become clear that stromal cells play an active role in tumor cell dissemination, which is caused by tumor-host interactions in which the microenvironment surrounding the tumor cells is an active partner during invasion and metastatic spread of cancerous cells [20,22-24]. Indeed, functional analysis of metastasis predictive signatures has indicated that these signatures likely also contain many genes that are specifically expressed in tumor stroma [9,10,25].

Although it has recently become clear that tumor stroma plays an important role in tumor invasion and metastasis, cancer research has traditionally focused on processes within tumor cells. Microarray studies generally only include tumor sections with a high percentage of tumor cells, thereby excluding a significant number of samples from signature analysis. To increase the number of patients that may benefit from newly developed diagnostic signatures, it is worthwhile to consider ways of designing signatures that also take into account tumor samples with low tumor cell percentages. Increased focus on stroma components will also likely improve our understanding of the mechanisms underlying tumorigenesis.

Head and neck squamous cell carcinomas (HNSCCs) arise in the upper aero-digestive tract and are the fifth most common malignancy in western populations, occurring with a rising frequency world-wide due to increased general life-expectancy and an increase in alcohol and tobacco consumption [26,27]. As with other tumor types, appropriate treatment depends on assessment of disease progression and, in particular, on assessment of the presence of metastases in regional lymph nodes close to the site of the primary tumor. Due to difficulties in detecting such (micro-)metastases reliably, a large number of patients do not currently receive the most appropriate treatment [28-30]. Several expression signatures have recently been reported for HNSCCs that can discriminate between metastasizing and benign tumors [25,31-33]. Although large-scale multi-center validation is still underway, assessment of independent samples indicates that implementation in clinical practice may improve treatment for up to 65% of patients with HNSCC in the oral cavity and oropharynx [25].

As with other solid-tumor profiling studies, one of the criteria for inclusion of samples in the latter study was the presence of more than 50% tumor cells in analyzed sections [13]. Here we investigate the influence of stroma/tumor percentage and show that the metastatic state of samples with lower tumor cell percentage is less accurately predicted. Using LCM to generate 35 related samples with artificially altered proportions of stroma versus tumor cells, the loss of predictive accuracy and the relationship between tumor cells and stroma is investigated further. The expression patterns of 685 metastasis associated genes are determined, leading to dissection of the metastatic signature into several components based on expression in tumor versus stroma and association with a metastatic or non-metastatic phenotype. The signature genes are very unevenly distributed over the different components, which has implications for our understanding of the metastatic process and for the design of expression signatures.

## Results

### Decrease in tumor cell percentage reduces predictive accuracy

**Figure 1:**
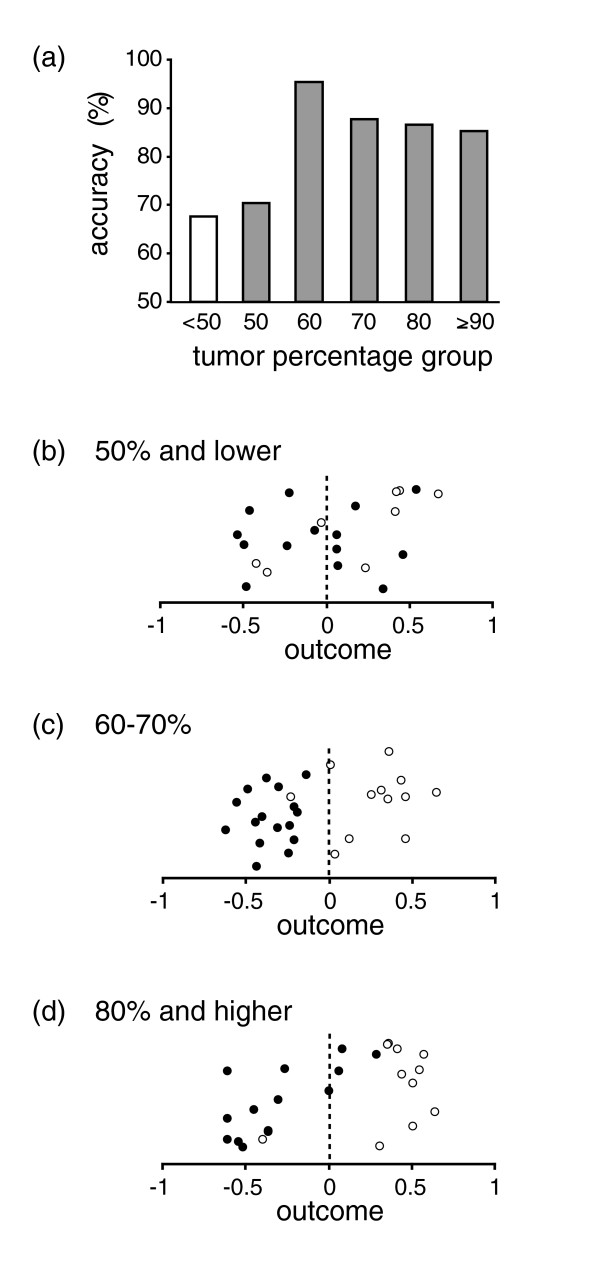
Predictive accuracy of the metastatic signature decreases for samples with low tumor percentage. **(a) **Predictive accuracy of the metastatic HNSCC signature per tumor percentage group. The predictive accuracy is expressed as the percentage of samples for which the previously published 120-gene primary tumor signature [25] correctly determined the absence or presence of metastasis based on comparison with histological examination of surgically removed neck lymph tissue. Signature outcome for samples with a tumor percentage of **(b) **50% or less, **(c) **between 60% and 70% and **(d) **80% or more. A signature outcome less than zero indicates a metastatic (N+) profile and an outcome above zero indicates a non-metastatic (N0) outcome. Solid circles indicate tumor samples from patients with metastasis; open circles indicate tumor samples from patients without metastasis.

HNSCC lymph node metastasis signatures have previously been identified using complete primary tumor sections that contain both tumor cells and tumor stroma [25]. Samples containing less than 50% tumor cells were excluded from this previous study, which resulted in identification of over 800 metastasis associated genes useful for prediction in a variety of signature compositions [34]. Within the samples included in these previous studies, a trend towards lower predictive accuracy for lower tumor percentage samples is indicated (Figure 1a, gray bars). This trend is even more apparent upon analysis of new samples with lower than 50% tumor cells (Figure 1a, white bar). Starting from the optimum tumor percentage of 60% to 70% (Figure 1c), the discriminatory power of the predictor is clearly reduced for samples containing less than 50% tumor cells (Figure 1b), which is in agreement with the decrease in predictive accuracy (Figure 1a). Interestingly, samples with the highest tumor percentage also show a slight loss of discriminatory power (Figure 1c), indicating that there may be an optimal composition of tumor sample sections for accurate prediction of the metastatic state. These results indicate a decrease in predictive accuracy that is related to an increased portion of stromal cells in tumor sections, despite the fact that metastatic signatures carry a considerable number of genes that are likely expressed in the stroma [9,34].

### Laser capture microdissection derived samples reveal a predictive bias

**Figure 2:**
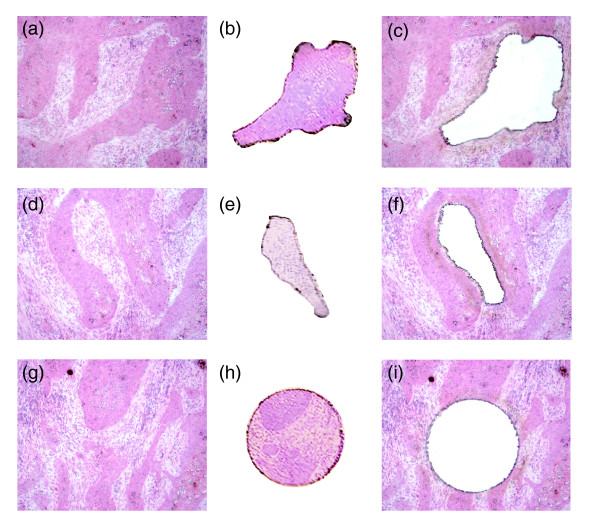
Isolation of tumor cells and tumor stroma from complete primary tumor sections. LCM microdissection was used to isolate tumor and stromal areas to generate artificial samples from complete primary tumor sections. **(a,d,g) **From primary tumor sections, areas comprising mainly **(b) **tumor cells or **(e) **tumor stroma, or **(h) **random circles were isolated using LCM. Samples with different tumor percentages were made by combining multiple tumor cell areas (b) and multiple tumor stroma areas (e) at varying ratios. Artificial samples for which the original tumor-stroma proportion was retained were made by isolation of multiple circled areas randomly distributed across the tumor section (h). See Materials and methods for more details. **(c,f,i) **Primary tumor sections after LCM of desired areas. The tissue sections shown here were colored using hematoxylin-eosin staining.

**Figure 3:**
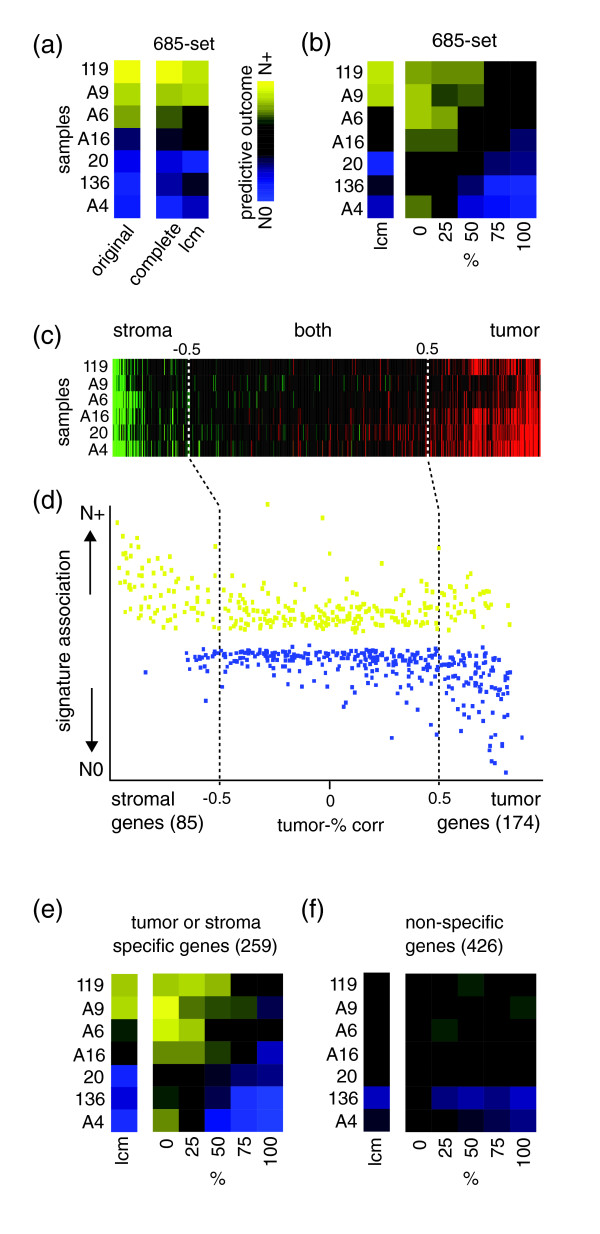
The HNSCC metastasis signature outcome shows tumor cell percentage bias due to skewed distribution of signature components. **(a) **Metastatic signature profiles of seven analyzed primary HNSCCs based on: complete tumor sections and the originally identified 102-signature genes [25] (original); complete sections and the set of 685 metastasis associated predictive genes (complete); and the 685-gene set and synthetic samples in which the original tumor-stroma proportion was retained (lcm). Blue indicates a non-metastatic (N0) profile, and yellow indicates a metastatic (N+) profile. **(b) **Metastatic signature profiles of synthetic samples from 7 primary tumors that retained the original tumor percentage (lcm) or contained 0%, 25%, 50%, 75% or 100% tumor cells, respectively. Profiles are based on the predictive 685 gene set; colors are as in (a). **(c) **The set of 685 predictive genes are ordered according to the correlation of their expression level with the 35 analyzed tumor percentages. Colors are based on a direct microarray comparison of tumor cells and tumor stroma, which confirms that negatively correlated (<-0.50) genes are mainly expressed in the stroma and positively correlated gene (>0.50) are tumor cell associated. Green indicates higher expression in tumor stroma compared to tumor cells and red indicates higher expression in tumor cells than in tumor stroma. Which of the 685 signature genes are distributed over which different components is described in detail in Additional data file 1. **(d) **Tumor percentage correlation and signature association (N0 or N+) of the predictive genes. Tumor percentage correlative groups as shown in (c). Blue indicates genes that are associated with the N0 signature profile, and yellow those associated with an N+ profile. Stromal genes are mostly N+ associated, that is, with higher expression in N+ primary tumor sections, while N0 profile related predictive genes are more commonly expressed in tumor cells, that is, down-regulated in N+ primary tumors. **(e) **As (b), but for the tumor and stromal specific predictive genes (259 genes). **(f) **As (b), but for the non-specific predictive genes that are similarly expressed between tumor cells and tumor stroma (tumor percentage correlation between -0.50 and 0.50).

Analysis of the influence of tumor cell percentage is confounded by the availability of sufficient samples representing a wide range of section compositions and, within each range of compositions, the availability of enough samples representing the possible predictive outcomes, that is, either with metastasis (N+) or without metastasis (N0). To circumvent this problem we applied LCM to generate, from complete primary tumor sections, multiple artificial samples that differed only in tumor percentage (see Figure 2 and Materials and methods for details). The samples chosen for this analysis represent a range of predictive accuracies for both the N0 and N+ outcome, including samples that are only marginally well predicted (Figure 3a, first column). A total of 35 artificial samples were generated by varying the proportion of tumor cells between 0% and 100%. The advantage of this approach is that any difference in signature profile between multiple artificial samples derived from a single tumor is entirely due to the different tumor percentages rather than individual sample heterogeneity. To determine whether this approach is valid, we first tested whether LCM samples that retained the original tumor percentage (Figure 2h) show the same signature outcome as the original complete tumor sections. The results of this analysis (Figure 3a, third column versus second column) confirm that generating artificial samples with LCM and implementation of the required additional RNA amplification procedure does itself not spoil the predictive outcome (Figure 3a).

From each of seven primary HNSCC tumor samples (three N0 and four N+, in which one N+ sample (A16) was weakly classified as N0), five artificial samples were created by combining isolated tumor (Figure 2b) and stromal areas (Figure 2e) in different proportions, thereby generating a total of 35 samples consisting of 0%, 25%, 50%, 75% or 100% tumor cells. Dye-swap replicate DNA microarray analysis was performed for these 35 samples and the HNSCC predictive signature outcome was tested using a predictor consisting of 685 genes. These were selected from a total of 825 metastasis associated genes [34] by removing genes that showed any bias in the double amplification procedure required for analysis of the small amounts of material available by LCM (see Materials and methods). Intriguingly, the predictive outcome was considerably influenced by tumor percentage (Figure 3b). This is especially true for samples with a low tumor content and agrees with the trend observed for the low tumor percentage sections shown in Figure 1a. Although differences between N0 and N+ tumors still remain, all seven analyzed tumors showed a bias towards a metastatic (N+) profile upon increase of the stroma percentage and a bias towards a non-metastasis (N0) profile upon increase in tumor cell percentage. Since this counterintuitive tumor percentage predisposition is likely caused by tumor or stroma cell specific gene expression, we decided to divide the signature genes into different categories and determine how the different components of the signature influenced the predictive outcome in a tumor percentage dependent manner.

### Metastasis is characterized by primary tumor gene expression loss and stromal cell activation

The first criterion for subdividing the metastasis associated genes was based on whether genes are expressed predominantly in stroma, in tumor cells or in both (Figure 3c). This subdivision into three subsets of genes is based on correlation of gene expression with the different tumor percentages in the entire set of 35 artificial samples, with genes ordered from left to right as stroma expressed and tumor expressed, respectively. To verify this subdivision, 100% tumor cell LCM samples were compared to 100% stroma LCM samples directly on 12 additional microarrays (dye-swap replicate for each of 6 samples for which there was still sufficient LCM material). The ratios of this direct comparison are depicted in green (stroma expressed) and red (tumor expressed) in Figure 3c and confirm the subdivision based on correlation with all the different tumor percentages. Interestingly, the results show that 12% of genes in the predictive signature are predominantly stroma expressed, 25% are more tumor cell specific, with the bulk equally expressed in tumor and stroma.

These three groups were then further subdivided into two categories each, based on whether up-regulation is associated with the presence or absence of metastasis (Figure 3d). Two striking observations become apparent upon subdividing the signature genes in this way. The first is the skewed distribution of genes over the six different categories. While there are a significant number of stroma expressed genes for which up-regulation is associated with the presence of metastasis, there are virtually no stroma expressed genes for which up-regulation is associated with the absence of metastasis (Figure 3d, left-hand side). In other words, the presence of metastasis is associated with up-regulation of a specific set of stroma expressed genes, but not with inactivation of stroma specific genes in the primary tumor. For the tumor cell expressed genes within the signature, an oppositely skewed distribution is also evident, although to a somewhat lower degree (Figure 3d, right-hand side). There are a significant number of tumor cell expressed genes for which increased expression is associated with the absence of a metastasis, but a much lower number of tumor cell expressed genes for which upregulation is associated with presence of metastasis. For HNSCCs in the oral cavity or oropharynx, the metastasizing primary tumor is, therefore, characterized by upregulation of stroma specific genes and inactivation of tumor cell specific genes. The 685 metastasis associated genes and their distribution over the different signature components are presented in Additional data file 1.

Besides providing important insights into the metastatic process itself, this skewed distribution may account for the predisposition of signature genes to falsely predict the presence of a metastasis for samples with reduced tumor percentage (Figure 3b). Because metastasis is associated with increased expression of a subset of stroma specific genes, with little to no down-regulation of stroma specific genes, an increased proportion of stroma in whole tumor sections will result in a bias towards an N+ prediction, even for primary tumors that are in fact N0. The other skew in the distribution, more down- than up-regulation of tumor cell specific genes in an N+ tumor, works in the same way and adds to the predisposition towards an N+ prediction in low tumor cell percentage samples. To test the idea that the skewed distribution underlies the bias towards predicting an N+ phenotype in samples with reduced tumor cell percentage, N0/N+ predictions were repeated on the 35 artificially composed LCM samples, using only those signature genes specifically expressed in either tumor cells or stroma. As expected, this signature is even more skewed towards predicting the N+ phenotype than the complete set of signature genes (Figure 3e versus Figure 3b).

### Skewed distribution of metastasis associated genes across distinct signature components

A second important observation that is apparent upon subdividing the signature genes into different categories can be made for genes that are expressed in both stroma and tumor (Figure 3d, middle group). Using only signature genes that are equivalently expressed in both stroma and tumor cells would be an ideal way in which to circumvent tumor cell percentage biases in signatures. Whereas hardly any skewed N0/N+ distribution is seen for this group, the predictive power to discriminate between N0 and N+ tumors is markedly reduced compared to the tumor cell and stroma specific genes. This is apparent from the lower degree of association with either an N+ or an N0 phenotype (Figure 3d). Because of their weaker association with either an N0 or N+ phenotype, a signature based exclusively on genes expressed in both tumor cells and stroma has insufficient predictive power to strongly discriminate between N0 and N+ primary tumors, either for the artificially generated samples (Figure 3f), or as tested on the entire original set of 66 primary tumor samples used to generate Figure 1 (overall accuracy is reduced from 86% to 76%).

**Figure 4:**
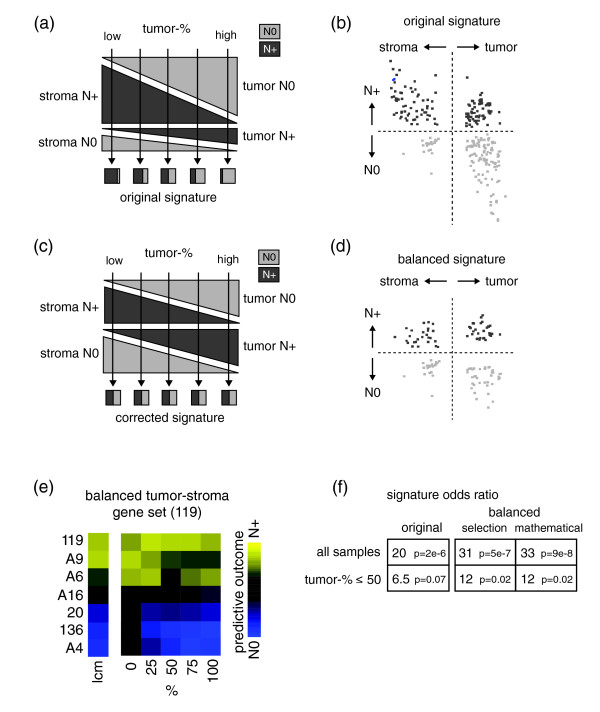
Balancing the tumor and stromal HNSCC signature components results in a more robust and accurate predictive profile. **(a) **Tumor cell specific and tumor stroma specific HNSCC signature genes can be dissected into four compartments: stroma N+, tumor N+, stroma N0 and tumor N0. Light grey indicates N0 association, and dark grey indicates N+ association. **(b) **Model for the relative contribution of the four components shown in (a) to the initial HNSCC signature. Combining the four components into one predictive outcome (indicated by arrows) results in tumor percentage signature bias. Low tumor percentage samples (left-hand side) show a more N+ biased profile (dark grey), whereas samples with a very high tumor percentage (right-hand side) exhibit a bias towards an N0 profile (light grey). **(c) **As (b), but for a corrected signature composition that does not exhibit a strong bias in the predictive outcome of low and high tumor percentage samples. **(d) **Selection of a set of 119 HNSCC signature genes that are equally distributed across the four different components, plotted similarly as in (a). **(e) **Predictive outcomes based on the corrected signature that consists of the 119 genes shown in (d). The corrected signature shows a strong reduction in predictive bias for samples with a low or very high tumor percentage; colors are as in Figure 3b. **(f) **Odds ratios for the signature outcome for prediction of metastasis based on the original signature, the balanced signature and through weighted correction based on the tumor cell percentage of samples.

Based on the results described above, the previously identified predictive HNSCC signature can be separated into one part that contains genes that are equally expressed between tumor and stroma but with limited predictive power, and a second part with tumor and stromal specific genes that have strong discriminatory power but a skewed N0/N+ distribution. A model for this composition and the ensuing bias in predictions shows the presence of four unequally distributed components (Figure 4a), alongside the actual distribution of such stroma and tumor cell specific genes (Figure 4b). The two large components contain N0 associated tumor genes (tumor N0) and N+ associated stromal genes (stroma N+). The two smaller components contain some tumor N+ genes and hardly any stroma N0 genes (Figure 4b). As is depicted (Figure 4a,b), the skewed sizes of these four components result in a signature that is unstable in its predictive outcome with regard to different tumor percentages (Figure 3e). If this model is accurate, adjustments to correct for overrepresentation should result in a predictive signature with reduced bias for different tumor percentages, as is indicated in the model shown in Figure 4c. Accordingly, from the initial comprehensive set of metastasis associated genes, a set of 119 predictive genes were selected that showed the greatest balance for the different signature components (Figure 4d; Additional data file 1). As expected, if these models are correct, the balanced HNSCC metastasis signature indeed shows a great reduction in tumor cell percentage bias for its predictive outcome when tested on the artificially composed LCM samples (Figure 4e). Using the balanced signature, the artificial tumor samples with a tumor percentage ranging from 25% to 100% now show a predictive outcome largely independent of tumor percentage and a strong reduction in the N+ predisposition for N0 samples containing no tumor cells (Figure 4e).

### Balanced signature performs better on low tumor cell percentage samples

To test whether predictive bias correction using a balanced signature does not exclusively work on the LCM composed samples, the performance of the balanced signature was determined on the set of 77 complete primary tumor sections (Figure 1), including the additional samples with less than 50% tumor cells. Here too, the balanced HNSCC metastasis signature outperforms the original signatures [34], especially for samples with a lower degree of tumor cells (Figure 4f). An odds ratio expresses the chance that the performance is based on random occurrence. The odds ratio for overall predictive accuracy for the less than 50% tumor cell samples rose from 6.5 (p = 0.07) to 12 (p = 0.02) upon application of the balanced signature. The improvement is incremental but significant for patients wishing to benefit from future diagnostic signatures, especially because this indicates that a larger group of samples can be included in signature profiling by taking into account the possibility of skewed distributions of signature genes. Another possible approach for adjusting the signature is weighting the predictive correlations of individual signature components based on tumor cell percentage in the sample. This mathematical correction results in a similar improvement in predictive accuracy (Figure 4f). Alternative methods for taking skewed signature compositions into account in future studies are discussed below.

## Discussion

In this study we have investigated the effects of tumor composition on the performance of a predictive signature, dissected the signature into different components and show that loss of predictive accuracy on low tumor cell percentage samples is, in part, caused by a skewed distribution of signature genes within these different components. The results have implications for our understanding of how metastases arise, for treatment of metastases and suggest several ways in which expression signatures can be improved.

### Stroma and tumor cell interactions

Functional category analyses of classifiers has previously indicated the presence of both tumor cell specific and stromal expressed genes in metastasis associated signatures [9,25,34]. By directly comparing LCM stroma fields with tumor fields we show that, for an exhaustive collection of 685 HNSCC lymph node metastasis associated genes, 12% are predominantly expressed in stroma, 25% in tumor cells and the majority in both tumor and stroma. This agrees with recent discoveries highlighting the contribution of the surrounding microenvironment towards cancer development [35-37] and the interplay between tumor and stromal cells that leads to metastasis [22,24,38].

A striking finding is the skewed distribution of stromal and tumor cell expressed genes with regard to their association with the presence or absence of metastasis (Figure 3d). Compared to the primary tumors that show no metastasis, the metastasizing primary head-neck tumor is characterized by exclusive up-regulation of a subset of stroma specific genes, concomitant with predominant inactivation of a subset of tumor specific genes. This is in agreement with the idea that tissue surrounding tumor cells is actively transformed into a metastasis supportive microenvironment [20,22,24]. The fact that metastasis is more strongly associated with down-regulation of tumor cell specific genes than their activation suggests that, in tumor cells, loss-of-function plays a more dominant role in acquiring a metastatic phenotype than gain of function. Future analyses may indicate whether any of the tumor cell metastasis associated genes are causal for the concomitant changes observed in stroma expression. Dissection of the large set of 685 metastasis associated genes [34] into much smaller groups of strongly metastasis associated genes with defined expression should simplify the task of finding suitable therapeutic targets for treatment of metastasis.

Two-thirds of the genes comprising the HNSCC metastatic signature have similar expression in tumor cells and stroma. On their own, these only marginally discriminate between N0 and N+ tumors, presumably due to lower differences in expression for these genes between the two tumor types. Because these genes are expressed in both stroma and tumor cells and exhibit less discriminatory power, such genes may be an indirect mark of genetic polymorphisms associated with the metastatic phenotype, rather than directly causal for metastasis. This idea is in line with indications that a metastasis expression signature is a product of genetic polymorphisms rather than changes caused during tumorigenesis [39]. Another interesting feature of the signature genes is the absence of highly specific, individual gene expression capable of discriminating between N0 and N+ tumor or stroma. This agrees with the difficulties in finding highly specific metastasis markers for primary tumors and the fact that successful signatures require contributions of large numbers of genes for accurate prediction. This also indicates that the metastatic phenotype is caused by relatively minor changes in expression of a large number of genes.

### Expression signature design

The skewed distribution of metastasis signature genes over the different components (Figure 3) has important implications for design of expression signatures. Samples consisting of lower than 50% tumor are generally excluded from profiling studies. This is an important but not well-documented issue. For example, approximately 30% of tumors in our current collection of head-neck tumor samples do not fulfill this criterion (P Roepman, unpublished results). Such samples have been excluded from many successful profiling studies and cannot be included in future implementation of diagnostic profiling unless approaches are devised to allow inclusion based on accurate predictions. Even a marginal decrease in tumor content to 40% or 25% for inclusion in future studies is a significant step forward for the patients involved.

Here we confirm that the metastatic status of samples with a lower proportion of tumor cells are indeed less accurately predicted (Figure 1) and demonstrate that, at least in part, this is due to the skewed distribution of metastasis associated genes over several different signature components (Figure 3). Because the most strongly metastasis associated genes are stromal genes that become up-regulated and tumor cell genes that are down-regulated (Figure 3d), the presence of a higher amount of stromal material will *a priori *predispose a metastatic signature to make an N+ prediction. The loss of discriminatory power observed on whole tumor sections is not always skewed towards making false N+ predictions for lower tumor percentage samples (Figure 1b), suggesting that other factors, such as sample heterogeneity, also play a role. Due to the large number of samples required to counter heterogeneity, it is, at present, not possible to determine unequivocally whether all the loss in predictive accuracy observed for lower tumor cell percentage samples (Figure 1a) can be attributed to the skewed distribution of signature genes. Nevertheless, the improved outcome on artificial LCM generated samples (Figure 4e) and complete tumor sections (Figure 4f) indicates that, if steps are taken to analyze signature compositions and correct for skewed distributions over the different components, then a larger number of patients will in future benefit from diagnostic signatures.

In this study, we present three methods for improved prediction of lower tumor percentage samples. The first method involves selection of signature genes expressed similarly in both tumor cells and stroma. The weaker discriminatory power of such genes is perhaps related to having no specific role in either tumor or stroma. When used on their own, the signature lacks sufficient discriminatory power, even when all 426 such genes are used together (Figure 3f). The two other approaches do include the skewed signature components, but compensate the resulting bias by selecting either a balanced number of genes (Figure 4d), or by tumor cell percentage weighted correction of individual component predictions. Both improve predictive accuracy for low tumor cell percentage samples, without loss of overall accuracy. Analysis of significantly more low-tumor-percentage samples is required to ascertain whether these are indeed the best approaches. Such a study could also investigate the possibility of designing two different independent signatures: one 'stromal-related' signature based on low tumor percentage samples and one 'tumor-related' signature based on high tumor percentage samples. Via this approach, a biological characteristic, that is, the interplay between tumor and stromal cells, will be divided into two separate signatures. Moreover, due to splitting the sample set into two, at least twice as many samples will be needed to achieve similar statistical significance. Insufficient numbers of such samples in our collection renders it as yet impossible to conclude whether this approach is feasible. Regardless of the issue of current sample availability, the importance of the present study is that it successfully dissects a clinically relevant diagnostic signature into separate components, and shows that skewed distribution of signature genes over the different components contributes to lower predictive accuracy for low tumor percentage samples. It will be important to determine whether other signatures have similar properties and future studies can now take the possibility of skewed distributions of signature genes into account, leading to inclusion of more samples and increasing the number of patients to which diagnostic signatures can be applied.

## Conclusion

Expression signatures that are derived from samples containing multiple tissue types can be dissected into multiple components. For a 685 gene signature associated with lymph node metastasis in HNSCC, there is a strikingly skewed distribution of the genes over the six different components of the signature. The metastasizing primary tumor is characterized by down-regulation of tumor cell specific genes and up-regulation of stromal genes. Dissection of the 685 metastasis associated genes in this way enables assessment of which gene products are better suited for targeted therapy. The skewed distribution of signature genes over the various components explains loss of predictive accuracy for samples containing lower amounts of tumor cells. The loss of predictive accuracy can, in part, be resolved by selecting genes that together form a signature with a balanced composition over the different components. This will allow more samples with lower amounts of tumor cells to be included in future analyses.

## Materials and methods

### Tumor samples

Previously determined gene expression data of 66 primary HNSCC tumor samples were used in this study [25]. In addition, 11 extra tumor samples were analyzed for their gene expression profile. Selection criteria for this additional set of samples were identical to the previous set of 66, except that complete tumor sections of these 11 samples showed a tumor content of less than 50%. RNA processing, microarray hybridization and analysis of the 11 samples was performed as previously described [25].

### Artificial tumor percentage samples

For 7 primary tumors (3 N0, 4 N+) selected from the previously analyzed set of 66 samples, 5 artificial samples were generated with 0%, 25%, 50%, 75% or 100% tumor cells and one artificial sample in which the original tumor percentage was retained. The artificial tumor percentage samples were generated by LCM of a tumor tissue section thus isolating 1 mm^2 ^tumor tissue in total. The artificial samples that differed in tumor percentage were made by combining multiple isolated tumor cell areas (Figure 2b) and multiple isolated tumor stroma fields (Figure 2e) in different ratios, for example, a 75% sample was generated by LCM of 0.75 mm^2 ^tumor cells and 0.25 mm^2 ^tumor stroma. The artificial samples in which the original composition was retained were generated by isolation of random circled areas from the complete tumor section (Figure 2h).

### LCM and RNA isolation

Frozen tumor sections (10 μm) were fixated on PALM MembraneSlides (PALM MicroLaser Systems, Bernried, Germany) and colored with hematoxylin for 30 seconds. LCM was performed using the PALM MicroBeam System. Total RNA from captured microdissected cells was isolated using the PicoPure™ RNA Isolation Kit (Arcturus, Sunnyvale, CA, USA). RNA quality was checked on the 2100 Bioanalyzer (Agilent, Santa Clara, CA, USA).

### RNA amplification and fluorescent labeling

RNA isolated from LCM samples was amplified using two rounds of T7 linear amplification. The first round was performed as described elsewhere [25] except that T7 *in vitro *transcription (IVT) was performed for two instead of four hours and without incorporation of aminoallyl-UTP. The first round cRNA was used as a template for a second round of amplification. Samples were vacuum concentrated to 9 μl and 1 μl random primers (1 μg/μl; Invitrogen, Paisley, Scotland) was added. Subsequently, first strand cDNA synthesis was performed as previously described [25] followed by incubation at 94°C for 5 minutes. After cooling the samples on ice, 1 μl of the previously used double anchored T7-poly(dT) primer was added [25] and the samples were incubated for 5 minutes at 70°C and subsequently for 3 minutes at 48°C. Second strand cDNA synthesis, second round IVT and cRNA cleanup were preformed as described elsewhere [25]. During the second amplification round, aminoallyl-UTP was incorporated into the generated cRNA, enabling direct coupling of fluophores before hybridization. Direct coupling of cy5 or cy3 fluophores was done as described previously [25]. Yield, quality and label incorporation were quantified spectrophotometrically and on the 2100 Bioanalyzer (Agilent).

### Gene expression analysis

Gene expression patterns were determined using 70-mer oligonucleotide DNA microarrays containing over 21,000 human gene features [25]. Before hybridization, the microarray slides were incubated in borohydrate buffer (2× SSC (0.3 M NaCl, 50 mM sodium citrate), 0.05% SDS and 0.25% w/v sodium borohydrate (Sigma-Aldrich, St. Louis, MO, USA) for 30 minutes at 42°C. We combined 300 ng of cy5 or cy3 labeled sample target (with a labeled nucleotide incorporation of 3% to 5%) with 300 ng reverse labeled reference cRNA [25], which was then fragmented using Ambion's Fragmentation kit (Ambion, Austin, TX, USA). Microarray hybridization was performed as described elsewhere [40]. The slides were scanned in the Agilent G2565AA DNA Microarray Scanner. Images were quantified and corrected for background using Imagene software (Biodiscovery, El Segundo, CA, USA). Quantified expression data were normalized as described previously [25]. Microarray layout, expression data and protocols have been deposited in compliance with MIAME in the ArrayExpress database, with accession numbers A-UMCU-3 and E-TABM-152.

### Metastasis signature outcome

The metastasis predictive signature outcome of each analyzed HNSCC sample was determined by calculating the correlation of its specific gene expression pattern with the previously determined typical metastatic (N+) and non-metastatic (N0) profiles, as described previously [25]. Combined, the N+ and N0 profile correlations denoted a single predictive signature outcome for each analyzed sample for a specific set of predictive genes. Positive correlation indicated an N+ profile, negative correlation an N0 profile. From the previously identified comprehensive set of 825 predictive genes [34], 685 genes that showed a robust profile when including the LCM and double amplification procedures were analyzed here. The removed 140 genes showed a bias in expression measurement due to the introduction of the LCM and double amplification procedures and gave a 1.5-fold difference in expression for at least 3 of the 7 analyzed tumor samples due to the changed technical procedures. The remaining 685 genes showed no or only marginal change in expression in 1 or 2 of the 7 analyzed tumor samples.

## Additional data files

The following additional data are available with the online version of this paper. Additional data file 1 is a table listing the 685 metastasis associated genes and their distribution over the different signature components.

## Supplementary Material

Additional file data 1 The 685 metastasis associated genes and their distribution over the different signature components Click here for file
